# Multifunctional NaNbO_3_: Photocatalytic Degradation of Methylene Blue and Intrinsic Antimicrobial Activity against *Enterococcus faecalis* and *Candida albicans*

**DOI:** 10.1021/acsomega.6c04846

**Published:** 2026-09-16

**Authors:** Daiane Fernandes, Marcelle M. Silveira, Thissiana da C. Fernandes, Cristiane W. Raubach, Janice L. Giongo, Rodrigo de A. Vaucher, Eduardo C. Moreira, Valmor R. Mastelaro, Sergio da S. Cava

**Affiliations:** † Post-Graduate Program in Materials Science and Engineering, 37902 Federal University of Pelotas, 96010-610, Pelotas, RS, Brazil; ‡ Post-Graduate Program in Microbiology and Parasitology, 37902 Federal University of Pelotas, 96160-000, Capão do Leão, RS, Brazil; § Post-Graduate Program in Engineering and Materials Science, Federal University of São Paulo, 12231-280, São José Dos Campos, SP, Brazil; ∥ Spectroscopy Laboratory, 186060 Federal University of Pampa, 96413-172, Bagé, RS, Brazil; ⊥ Physics Institute, São Paulo University, 13560-970, São Carlos, SP, Brazil; # Post-Graduate Program in Physics, 37902 Federal University of Pelotas, 96160-000, Capão do Leão, RS, Brazil

## Abstract

In this paper, sodium niobate (NaNbO_3_) was synthesized by the microwave-assisted hydrothermal method at 150 °C for 70 min, followed by calcination at 550 °C for 2 h. X-ray diffraction revealed the coexistence of orthorhombic *P*21*ma* and *Pbma* phases, while scanning electron microscopy showed a one-dimensional rod-like morphology with an average diameter of about 200 nm. The material exhibited a specific surface area of 5.45 m^2^/g, a pore volume of 0.0269 cm^3^/g, and an average pore diameter of 20.52 nm. X-ray photoelectron spectroscopy indicated the presence of defect-related species, while photoluminescence emissions in the blue and green regions provided complementary evidence for defect-related electronic states. The optical band gap, estimated by diffuse reflectance spectroscopy, was approximately 3.56 eV. Mott–Schottky analysis indicated n-type semiconductor behavior and flat-band potential of −0.76 V vs Ag/AgCl. Electrochemical impedance spectroscopy showed improved interfacial charge-transfer behavior under light irradiation. The material exhibited efficient photocatalytic activity, achieving complete degradation of methylene blue (MB) under UV irradiation, with good stability after five successive reuse cycles. Radical scavenger experiments revealed that h^+^, ·OH, and ·O_2_^–^ species are active in the photocatalytic mechanism. The synthesized NaNbO_3_ demonstrated effective antimicrobial activity against *Enterococcus faecalis* and *Candida albicans*, completely inhibiting microbial growth at concentrations ≥1.25 mg/mL. To the best of our knowledge, this is the first evidence of antimicrobial activity against these microorganisms using NaNbO_3_, highlighting its potential for environmental and biomedical applications.

## Graphical abstract

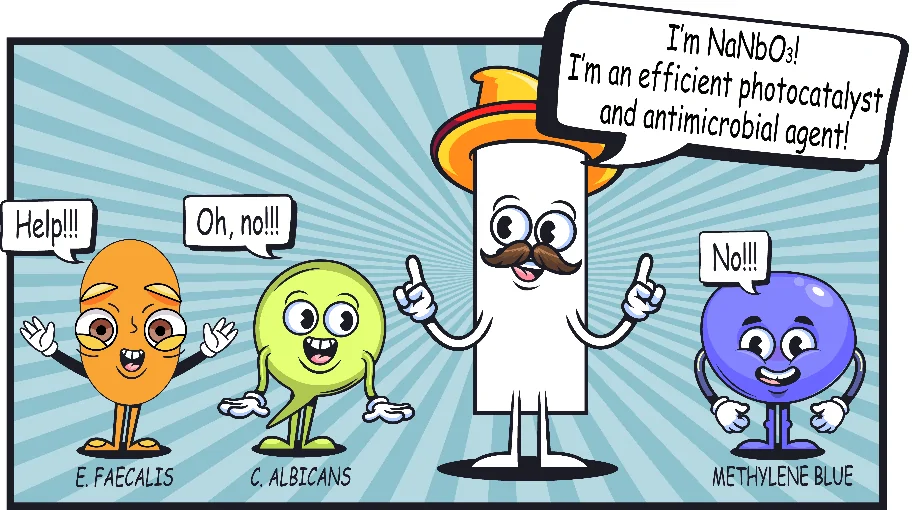

## 1 Introduction

Water pollution is one of the most pressing environmental issues, given the essential role of this natural resource in sustaining life on Earth. Among the major contaminants, recalcitrant pollutants such as synthetic dyes are particularly concerning. One representative example is methylene blue (MB), which is widely used in various industrial sectors. It exhibits fluorescent properties and a high affinity for cellular membranes, making it useful in biological and diagnostic applications. Although MB has demonstrated several beneficial medicinal effects, these occur only under controlled and safe conditions, in contrast to exposure or ingestion through contaminated water. Such exposure can lead to several adverse health effects, in addition to harming aquatic and plant organisms.^1,2^ Therefore, the removal of this pollutant from water is essential, and heterogeneous photocatalysis stands out as an efficient approach.

The photocatalytic mechanism is based on the excitation of electrons from the valence band (VB) to the conduction band (CB) of a semiconductor by photons with sufficient energy, generating electron/hole pairs (e^–^/h^+^).^3^ These photogenerated charge carriers promote the degradation of various pollutants, including MB.^3–5^ However, limitations such as rapid recombination of e^–^/h^+^ pairs and low stability restrict the practical use of many semiconductors, motivating the search for materials with improved performance.

In this context, sodium niobate (NaNbO_3_), although initially explored in the field of piezoceramics, has emerged as a promising photocatalyst. It has shown efficient photocatalytic activity in the degradation of organic pollutants, including antibiotics, 2,4-dichlorophenol, and dyes.^6–9^ This material exhibits high physicochemical stability, corrosion resistance, low toxicity, and water insolubility, characteristics that make it advantageous for photocatalytic applications. Furthermore, NaNbO_3_ can be obtained with a one-dimensional (1D) morphology, which has shown superior photocatalytic performance compared to its isotropic counterparts, as reported for the degradation of rhodamine B and red X-GRL dyes, as well as for H_2_ evolution.^8,10^

Despite the growing interest in antimicrobial materials, the use of NaNbO_3_ for this purpose remains poorly explored. Previous studies have associated its antimicrobial activity with the generation of reactive oxygen species under external stimuli, such as catalytic activation or polarization, leading to bacterial inactivation.^11–14^ In contrast, aiming at less complex and more practical applications, we recently reported the first evidence of the intrinsic antibacterial activity of NaNbO_3_ against *Escherichia coli*, *Staphylococcus aureus*, and *Klebsiella pneumoniae*.^15^

The extension of this investigation is particularly relevant in the case of *Enterococcus faecalis* (*E*. *faecalis*). This bacterium exhibits remarkable resistance to adverse environmental conditions, a strong biofilm-forming ability, and reduced susceptibility to several antibiotics. Human exposure has been associated with gastrointestinal, skin, and mucosal infections. In addition to its clinical relevance, it is widely employed as an indicator of fecal contamination in water due to its high prevalence in sewage and persistence in aquatic environments.^16–19^

Additionally, the antifungal activity of NaNbO_3_ has not yet been explored, representing a significant research gap, especially considering that fungal infections are responsible for millions of deaths annually worldwide. *Candida albicans* (*C*. *albicans*) stands out as the main etiological agent of candidiasis and the predominant cause of fungal infections in humans. This dimorphic fungus is part of the normal microbiota of the skin and mucosal surfaces in approximately 60% of the human population, but it can transition to a pathogenic state under immunosuppressive conditions. The relevance of this opportunistic microorganism lies in its ability to cause invasive infections through dissemination via the bloodstream and subsequent colonization of internal organs. Furthermore, the limited efficacy of conventional antifungal treatments has contributed to increased mortality rates and healthcare costs. Given these concerns, the World Health Organization has included *C*. *albicans* in the list of priority fungal pathogens.^20,21^

Considering the above, 1D NaNbO_3_ was synthesized in this study using a microwave-assisted hydrothermal method. This approach offers advantages such as rapid and volumetric heating, making it an effective route for material synthesis. The obtained material exhibited efficient photocatalytic performance toward MB degradation, along with good reusability. In addition, the material demonstrated effective antimicrobial activity against *E*. *faecalis* and *C*. *albicans*, completely inhibiting microbial growth. To the best of our knowledge, this is the first report describing the activity of NaNbO_3_ against these microorganisms. Given the increasing demand for multifunctional materials, these findings highlight the potential of NaNbO_3_ for environmental and biomedical applications.

## 2 Experimental Section

NaNbO_3_ was synthesized using the microwave-assisted hydrothermal method, adapted from a previous study.^15^ An aqueous sodium hydroxide solution (8.33 M) containing 0.00376 mol of niobium pentoxide (donated by CBMM) was prepared and magnetically stirred at room temperature. The suspension was at 150 °C for 70 min, using a heating rate of 5 °C/min. The resulting solid was washed several times with distilled water until reaching neutral pH, dried at 90 °C overnight, and calcined at 550 °C for 2 h.

X-ray diffraction (XRD) analysis was carried out using a diffractometer (Ultima IV, Rigaku) with Cu–Kα radiation (λ = 1.5406 Å) at 40 kV and 30 mA. Scanning electron microscopy (SEM) was performed with a field emission scanning electron microscope (Supra 35, Zeiss) operating at 20 kV. The textural properties were evaluated by N_2_ adsorption–desorption measurements at 77 K using a surface area analyzer (Gemini VII 2390A, Micromeritics). The specific surface area was determined using the Brunauer–Emmett–Teller (BET) method, while the pore volume and average pore diameter were determined using the Barrett–Joyner–Halenda (BJH) method. X-ray photoelectron spectroscopy (XPS) was performed using a spectrometer (ESCA+, SCIENTIA OMICRON) with a high-performance hemispherical analyzer (EAC2000) and monochromatic Al–Kα radiation (hν = 1486.6 eV). The operating pressure in the ultra-high vacuum chamber (UHV) was around 10–9 Pa during the analysis. The XPS high-resolution spectra were recorded at a constant pass energy of 30 eV, with a 0.05 eV step. A charge neutralizer (CN10) was used to exclude the surface charging effects. UV–Vis diffuse reflectance spectrum was recorded with a UV–Vis spectrophotometer (HR2000+, Ocean Optics), and the optical band gap was estimated using the Wood–Tauc method. The photoluminescence (PL) was analyzed using a fluorescence spectrometer (Cary Eclipse, AGILENT), with an excitation wavelength of 260 nm. Electrochemical measurements were performed in a conventional three-electrode configuration using 0.1 M NaOH as the electrolyte. The NaNbO_3_ film was prepared by drop-casting a suspension onto a fluorine-doped tin oxide (FTO) substrate, which served as the working electrode. A Pt wire and an Ag/AgCl (3 M KCl) electrode were used as the counter and reference electrodes, respectively. Electrochemical impedance spectroscopy (EIS) measurements were performed under AM 1.5G illumination (100 mW cm^–2^) over a frequency range of 100 kHz to 0.1 Hz using an AC amplitude of 10 mV at the selected applied potential. Mott–Schottky (MS) measurements were conducted in the dark using EIS at a frequency of 1 kHz with an AC perturbation amplitude of 10 mV.

The photocatalytic experiments were carried out under UV light using six lamps (15 W each, λ_max_ = 254 nm). The photocatalyst was dispersed in 50 mL of MB solution and first stirred in the dark for 30 min to establish adsorption–desorption equilibrium. Adsorption experiments were conducted and the results are presented in Fig. S1. Under light irradiation, aliquots were collected at specific time intervals and their absorbance was measured using a UV–Vis spectrophotometer (SP200 UV, BEL PHOTONICS). The removal efficiency was determined from the C/C_0_ ratio, where C_0_ and C are the initial and residual dye concentrations, respectively. Scavenger experiments were performed using disodium ethylenediaminetetraacetate (EDTA, hole (h^+^) scavenger, 1 mM), isopropyl alcohol (IPA, hydroxyl radical (·OH) scavenger, 100 mM), and p-benzoquinone (BQ, superoxide radical (·O_2_^–^) scavenger, 1 mM). For reusability tests, the photocatalyst was recovered by centrifugation, dried, and reused. The experiment was carried out in duplicate.

For the evaluation of antimicrobial activity, NaNbO_3_ was initially dispersed in dimethyl sulfoxide and incorporated into the agar. For antibacterial assays, Mueller–Hinton agar was used, while Sabouraud dextrose agar was employed for antifungal assays. The prepared media were immediately dispensed into sterile Petri dishes. Fresh inocula of *E*. *faecalis* and *C*. *albicans* were obtained from single isolated colonies grown on their respective media. The cells were suspended in sterile saline and adjusted to approximately 0.5 McFarland standard. The standardized inocula were then subcultured onto Petri dishes containing media supplemented with the different concentrations of NaNbO_3_ and incubated at 37 °C for 24 h (*E*. *faecalis*) and 48 h (*C*. *albicans*). The control groups consisted only of agar (negative control) and inoculated media without NaNbO_3_ (positive control). After the incubation period, colony-forming units (CFU) were counted and expressed as log CFU/mL. Data are presented as mean ± standard deviation from triplicate determinations. Statistical analysis was performed using GraphPad Prism 6 software. Means were compared using two-way analysis of variance (ANOVA), followed by Dunnett’s multiple comparison test. Differences were considered statistically significant at p < 0.05.

## 3 Results and Discussion

The XRD pattern (Fig. 1a) shows the coexistence of two orthorhombic phases, P21ma and Pbma (ICSD 74642 and ICSD 97669, Fig. 1b). The structural refinement using the Rietveld method is provided in the Supplementary Material (Fig. S2 and Table S1). Low-temperature crystallization (approximately 600 °C) favors the formation of the non-centrosymmetric P21ma phase, characterized by more distorted NbO_6_ octahedra that result in a polar structure. With the increase in temperature (below 950 °C), these distortions are reduced and a structural reorganization occurs, resulting in the centrosymmetric and nonpolar Pbma phase.^22^ However, even in samples crystallized at low temperatures, the presence of a centrosymmetric fraction within the non-centrosymmetric matrix is commonly observed.^23,24^ Non-centrosymmetric materials exhibit intrinsic internal electric fields arising from their asymmetric crystal structure, which can enhance the separation of e^–^/h^+^ pairs and suppress their recombination.^25^ Therefore, the predominance of the P21ma phase may contribute positively to the photocatalytic activity of the synthesized NaNbO_3_.

**Figure 1:**
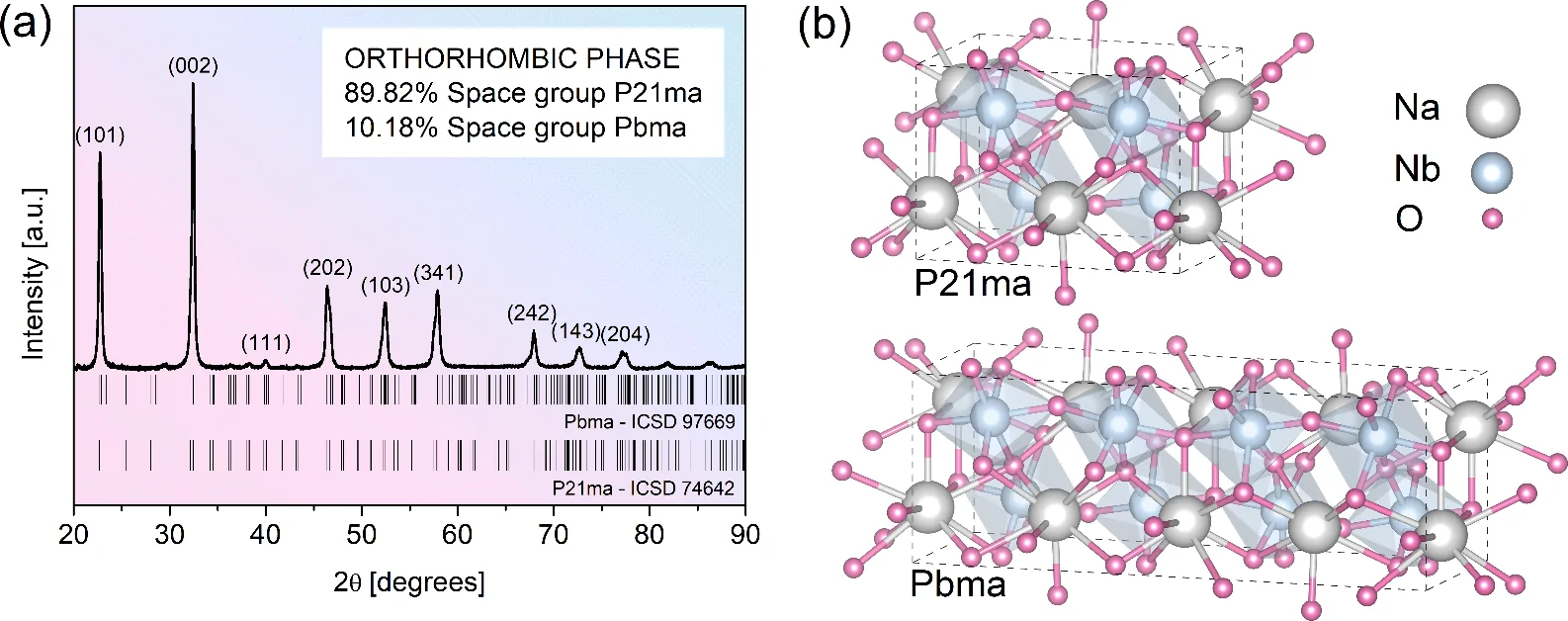
(a) XRD pattern and (b) Structural representations of the orthorhombic P21ma and Pbma phases.

The SEM image (Fig. 2) reveals that the prepared sample exhibits a rod-like shape, with an average diameter of approximately 200 nm and lengths of several tens of micrometers. This 1D morphology originates from the initial formation of the metastable Na_2_Nb_2_O_6_·H_2_O phase, which exhibits a fibrous shape. After dehydration, the nucleation process of NaNbO_3_ crystals begins. When this nucleation occurs under a short-range diffusion regime, the 1D structure is preserved.^26^ Such morphology is particularly relevant for catalytic applications because its high aspect ratio favors the availability of active sites.^27,28^ Additionally, 1D nanostructures have attracted considerable attention for antimicrobial applications.^29,30^

**Figure 2:**
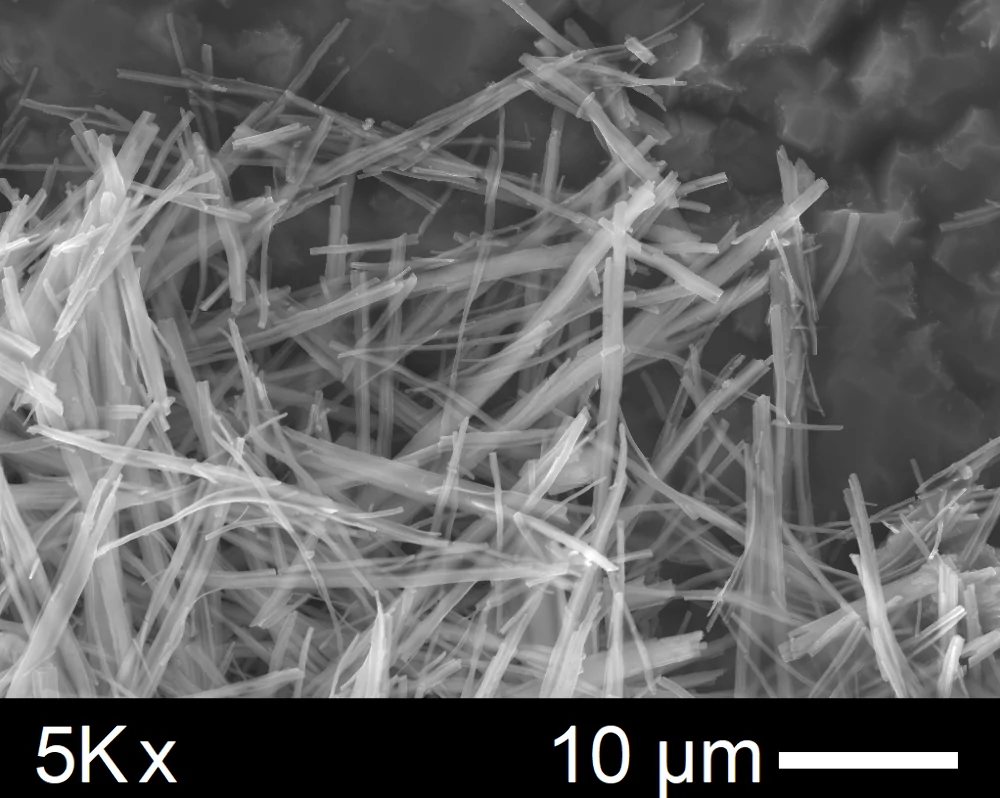
SEM image (5.000× magnification).

The BET analysis revealed a specific surface area of 5.4480 m^2^/g. Although modest, this value exceeds those commonly reported for NaNbO_3_ with isotropic morphologies (0.5–2.0 m^2^/g).^31–33^ The relatively higher specific surface area maximizes the interface between the material and the surrounding medium, which is advantageous for both photocatalytic and antimicrobial applications. Additionally, the BJH analysis indicated a pore volume of 0.0269 cm^3^/g and an average pore diameter of 20.52 nm, characterizing the material as mesoporous.^7^ Mesoporous materials are known to facilitate the diffusion and adsorption of dye molecules, thereby improving their accessibility to the catalyst surface and benefiting photocatalytic reactions.^34,35^

The surface chemical composition was investigated by XPS, and the survey spectrum (Fig. 3a) confirms the presence of Na, Nb, and O elements. The high-resolution Na 1s XPS spectrum (Fig. 3b) exhibits a peak centered at 1071.3 eV, corresponding to the Na^+^ oxidation state.^12^ An additional component at slightly lower binding energy suggests the presence of Na atoms in distinct chemical environments, possibly associated with local structural distortions or oxygen vacancies.^36,37^ The Nb 3d spectrum (Fig. 3c) exhibits a typical spin–orbit doublet with peaks centered at 206.9 eV (Nb 3d_5/2_) and 209.7 eV (Nb 3d_3/2_), characteristic of Nb^5+^.^7,11,12^ Additional low-intensity components at lower binding energies suggest the presence of Nb^4+^ species, associated with oxygen deficiency and structural defects.^38,39^ In the O 1s spectrum (Fig. 3d), three contributions are observed. The peaks centered at 529.7, 532.4, and 534.2 eV correspond to the lattice oxygen, oxygen vacancy, and chemisorbed oxygen on the sample surface, respectively.^11,12,40^ Overall, the results suggest that the synthesized NaNbO_3_ exhibits a defect-rich surface. Such defects may introduce localized electronic states that can act as charge-trapping centers, delaying the recombination of photogenerated e^–^/h^+^ pairs. Additionally, oxygen vacancies can serve as active sites for O_2_ adsorption and activation, promoting the formation of ·O_2_^–^, species that can participate in photocatalytic reactions.^27,35,38^ ^,^ ^40,41^ Defect-rich oxide surfaces have also been associated with enhanced intrinsic antimicrobial activity, highlighting the important role of surface defects in mediating microorganism–material interactions.^42–44^

**Figure 3:**
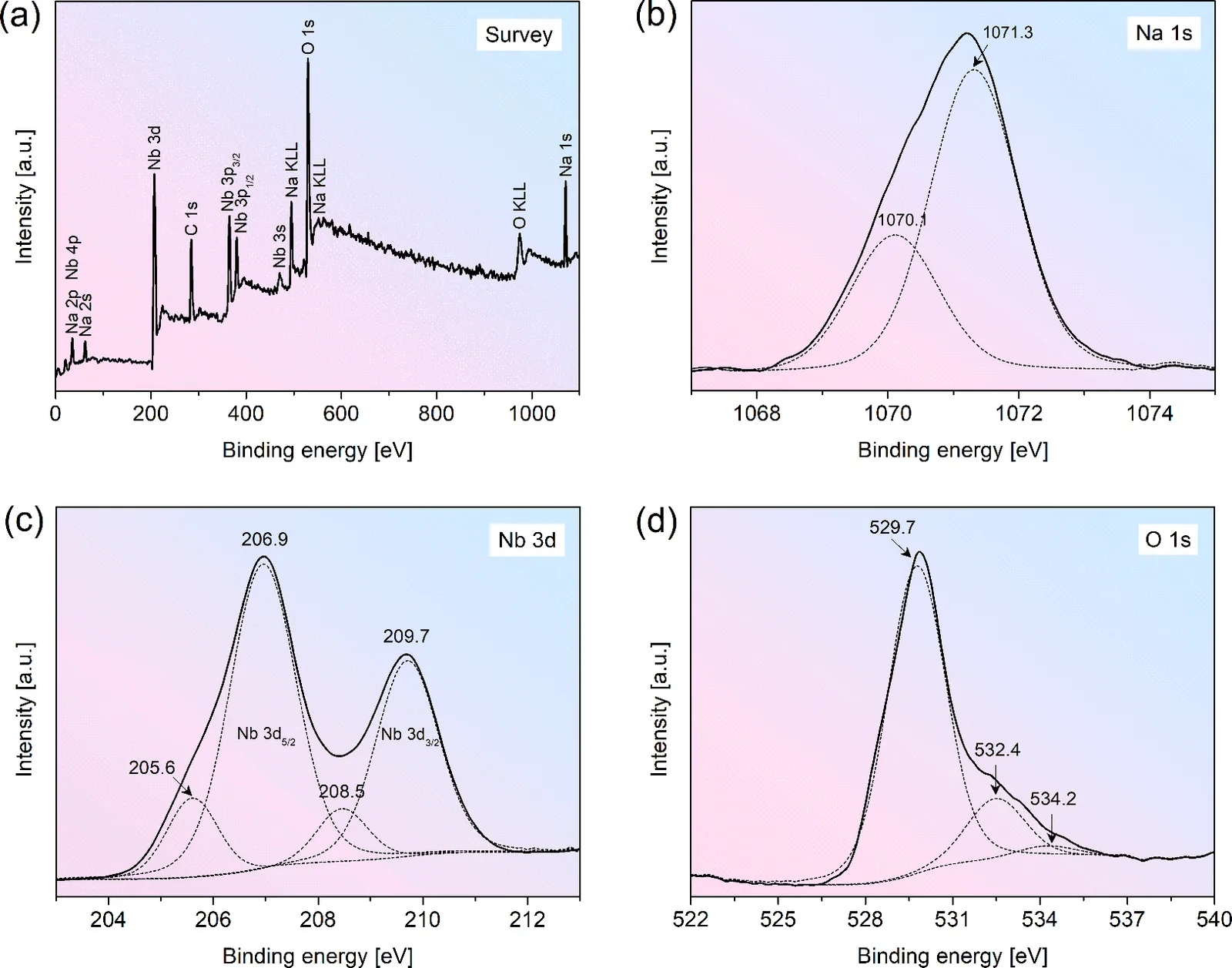
(a) XPS survey spectrum (b) high-resolution XPS spectra of Na 1s, (c) Nb 3d, and (d) O 1s.

The band gap was estimated at approximately 3.56 eV (Fig. 4a), in agreement with the literature.^11,12,22^ This energy indicates that NaNbO_3_ is predominantly activated by UV radiation. Consequently, its photocatalytic efficiency under natural sunlight may be limited by the low UV fraction of the solar spectrum, a characteristic commonly observed for wide-band-gap semiconductors. Gouget et al.^22^ reported that the orthorhombic symmetries P21ma and Pbma of NaNbO_3_ exhibit similar electronic structures, with the CB and VB mainly composed of Nb 4d and O 2p orbitals, respectively. Therefore, the main absorption edge is associated with electronic transitions between these orbitals. A slight shoulder near the absorption edge is also observed in the Tauc plot, suggesting defect-related sub-band-gap absorption. This behavior is consistent with the XPS results (Fig. 3), which indicate the presence of structural defects capable of introducing electronic states within the band gap.

**Figure 4:**
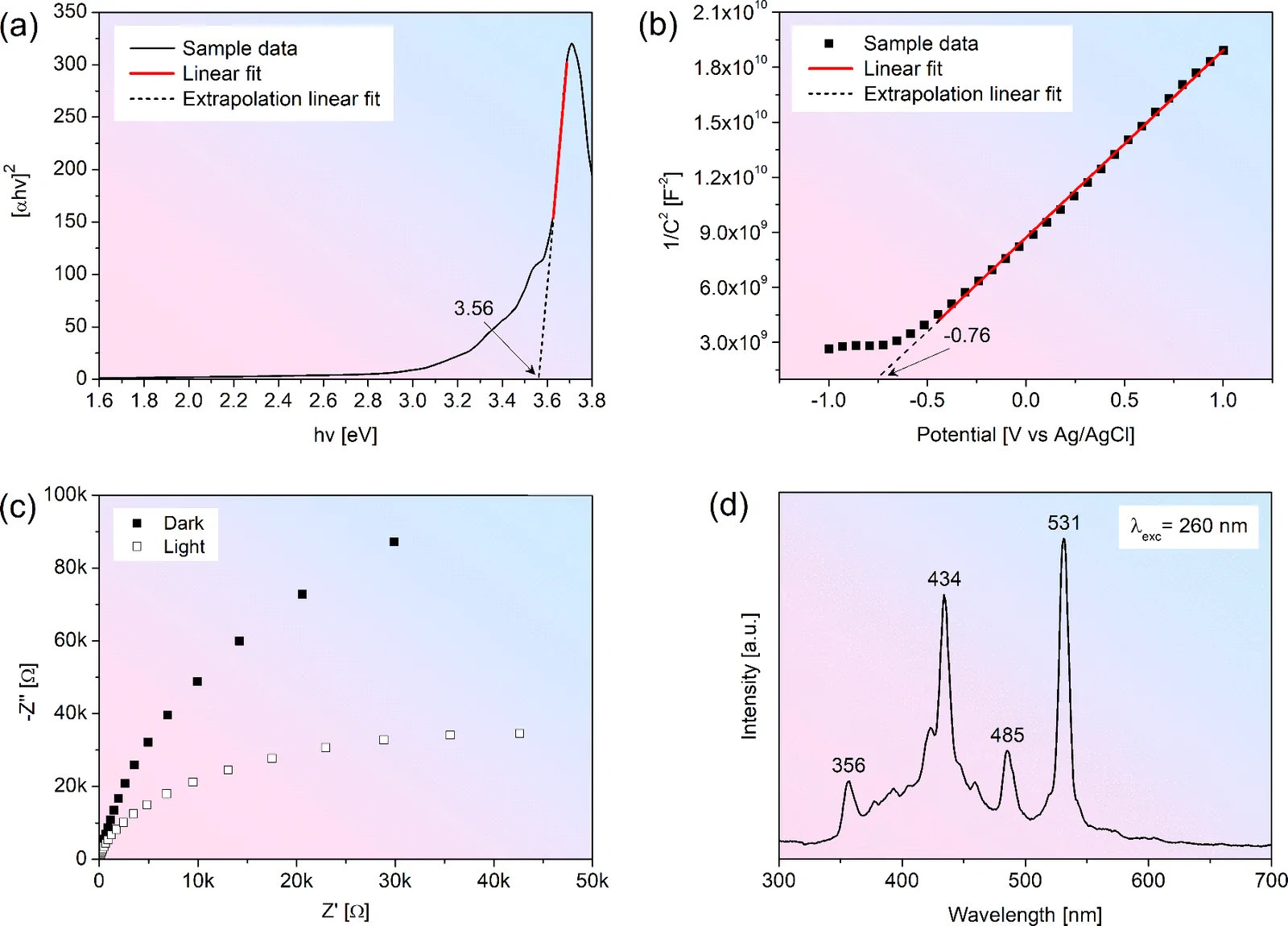
(a) Tauc plot for estimation of the band gap energy, (b) Mott–Schottky plot, (c) Nyquist plots under dark and light conditions, and (d) PL emission spectrum recorded at room temperature.

Fig. 4b shows the Mott–Schottky plot of NaNbO_3_. The positive slope confirms the typical behavior of an n-type semiconductor. By extrapolating the linear region of the curve (R^2^ = 0.9984), a flat-band potential of −0.76 V vs Ag/AgCl was obtained, corresponding to approximately −0.55 V vs NHE. For n-type semiconductors, the CB edge is generally considered to lie approximately 0.10 V more negative than the flat-band potential.^45^ Accordingly, the CB edge was estimated to be approximately −0.65 V vs NHE, consistent with values reported in the literature.^46–48^ Considering the band gap energy of 3.56 eV (Fig. 4a), the VB edge was estimated to be approximately +2.91 V vs NHE. The band-edge positions indicate sufficient thermodynamic potential for the reduction of molecular oxygen to superoxide radicals (O_2_/·O_2_^–^, −0.33 V vs NHE) and the oxidation of water molecules to hydroxyl radicals (H_2_O/·OH, +2.40 V vs NHE).^5,48^ Therefore, NaNbO_3_ exhibits energetically favorable conditions for the generation of reactive oxygen species (ROS) under light irradiation, which can promote the oxidation and reduction reactions involved in the photocatalytic process.

Fig. 4c shows the Nyquist plots under dark and illuminated conditions. A significant decrease in the impedance response is observed under illumination, indicating a reduction in the charge-transfer resistance at the semiconductor/electrolyte interface. This behavior is associated with more efficient interfacial transfer and separation of photogenerated charge carriers.^40,45,49,50^ As a result, the reduced probability of e^–^/h^+^ pairs recombination increases their availability to participate in reduction and oxidation reactions at the NaNbO_3_ surface, thereby favoring photocatalytic processes.

Figure 4d shows the PL emission. The emission at 356 nm corresponds to an energy of approximately 3.48 eV, which is close to the band gap (Fig. 4a), and can therefore be associated with near-band-edge recombination. In contrast, emissions at 434, 485, and 531 nm occur at lower energies, indicating the involvement of intermediate electronic states in the radiative transitions. The green emission is associated with deeper defect states, mainly related to oxygen vacancies and structural distortions, while the blue emission has been attributed to distortions of the NbO_6_ octahedra.^51,52^ Although higher PL intensity is frequently associated with greater recombination of e^–^/h^+^ pairs in comparative analyses of different materials or samples, such an interpretation cannot be established in the present study.^31,33,46^ ^,^ ^48,50^ Thus, the PL results provide complementary spectroscopic evidence for the presence of defect-related electronic states in the synthesized NaNbO_3_.

Figure 5a shows the removal of MB using different NaNbO_3_ loadings and initial dye concentrations, together with the corresponding photolysis controls, including a 30 min dark period prior to light irradiation. MB adsorption was approximately 42.4% and 71.6% for 25 and 50 mg of NaNbO_3_, respectively, at 5 mg/L, while values of 10.7% and 43.7% were obtained at 10 mg/L. Upon light irradiation, the MB concentration progressively decreased in all systems containing NaNbO_3_ until complete removal was achieved. Although the lower photocatalyst loading resulted in considerably less MB adsorption, both 25 and 50 mg of NaNbO_3_ achieved complete MB removal after 30 min of irradiation at 5 mg/L and after approximately 50 min at 10 mg/L. This behavior indicates that, despite the different amounts of MB remaining in solution at the onset of irradiation, the lower NaNbO_3_ loading was still sufficient to achieve the same final removal as the higher loading, demonstrating its effective photocatalytic activity. In contrast, the substantially lower degradation observed in the absence of photocatalyst indicates that direct photolysis makes only a minor contribution under the employed conditions, further supporting the photocatalytic role of NaNbO_3_ in MB degradation.

**Figure 5:**
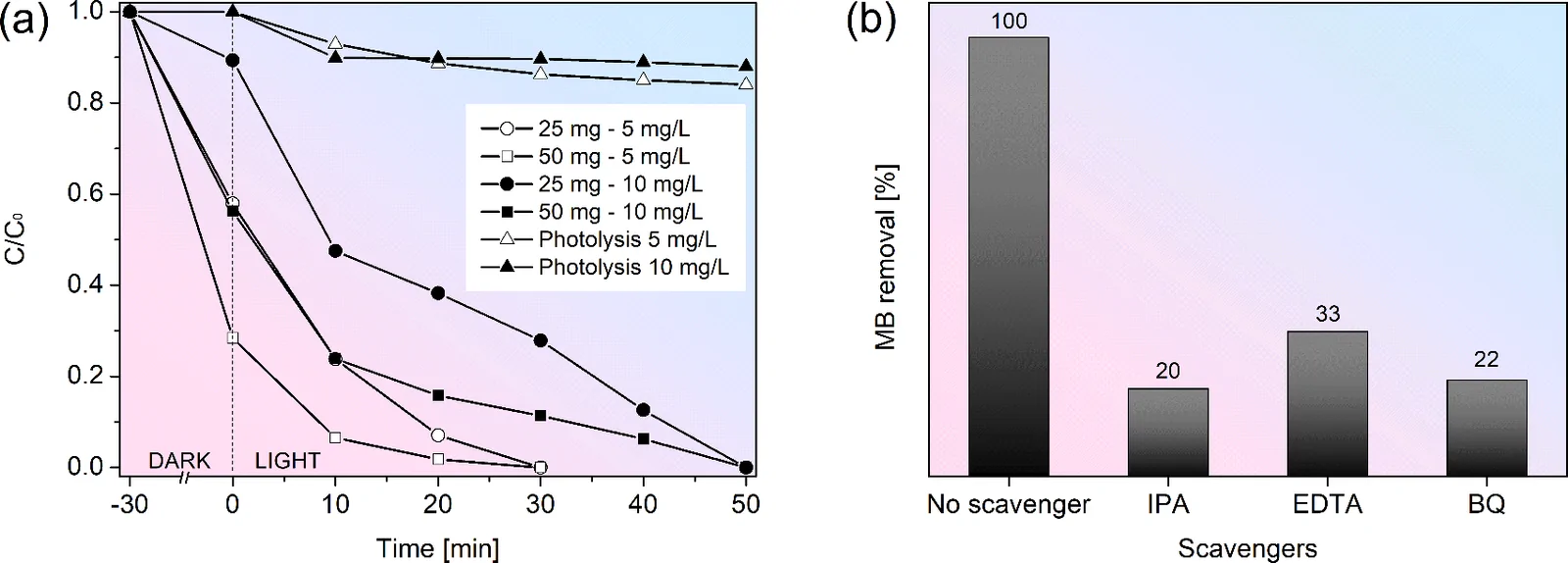
(a) Removal of MB using different NaNbO_3_ loadings and initial dye concentrations, photolysis controls, and the dark adsorption period and (b) Scavenger experiments using IPA, EDTA, and BQ, performed with 50 mg of NaNbO_3_ and an initial MB concentration of 10 mg/L after 50 min of light irradiation.

Scavenger experiments performed using 50 mg of NaNbO_3_ and an initial MB concentration of 10 mg/L showed that the addition of EDTA, IPA, and BQ reduced the dye removal efficiency to 33%, 20%, and 22%, respectively (Fig. 5b). These results suggest the involvement of h^+^, ·OH, and ·O_2_^–^ in the MB degradation process. However, this approach provides indirect evidence of reactive species participation and does not allow their relative contributions to the photocatalytic process to be quantitatively determined.^53^ Based on these results, together with the Tauc plot (Fig. 4a) and Mott–Schottky analysis (Fig. 4b), a possible photocatalytic mechanism for MB degradation using NaNbO_3_ is proposed in Fig. 6. Additionally, a reusability test was conducted under the same experimental conditions, and the efficiency remained similar after five consecutive cycles, suggesting good stability of the material.

**Figure 6:**
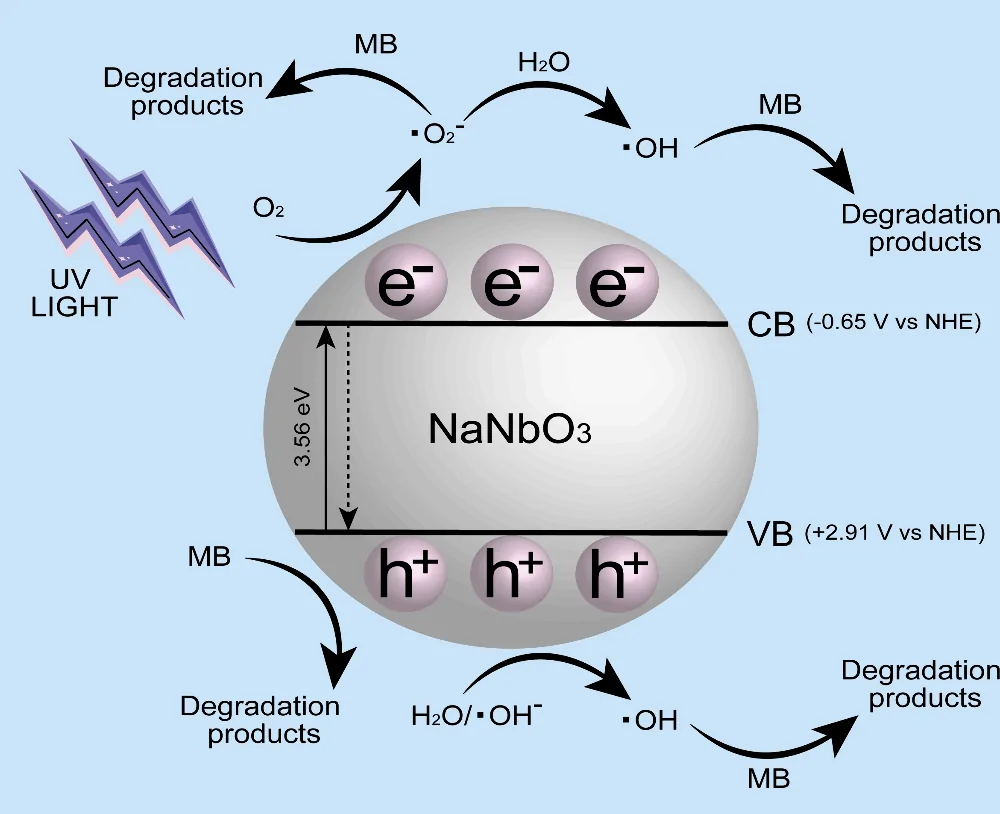
Schematic representation of the proposed photocatalytic mechanism for the degradation of MB dye using NaNbO_3_.

Table 1 summarizes representative studies reporting the photocatalytic degradation of MB using different photocatalysts. Although direct comparisons with these systems are not appropriate due to differences in the experimental conditions, the prepared NaNbO_3_ exhibited efficient photocatalytic activity under the investigated conditions, demonstrating its potential for the removal of this pollutant.

**Table 1:** Comparison of Photocatalytic Performance Reported for Different Photocatalysts

Photocatalyst	Catalyst mass per volume^a^ [mg/mL]	Concentration MB [mg/L]	Time [min]	Removal [%]	UV light^a^ [W]	Ref.
NaNbO_3_	0.5/1	10	50	100	90	This work
ZnO	1/1	10	60	80	250	^54^
TiO_2_ P25				70		
ZnO/NaNbO_3_				97		
NaTaO_3_	1/1	10	180	47	250	^55^
Nb-doped NaTaO_3_				95.7		
TiO_2_	2/1	3	240	56	8	^56^
TiO_2_/ZnO				70		
Ag-doped TiO_2_/ZnO				83.6		
Al-doped TiO_2_/ZnO				60		
CaTiO_3_	0.5/1	8.546	330	97	11	^57^
Ni-doped TiO_2_/ZnO	0.2/1	3.1985	160	85.5	NE	^58^
Mg-doped TiO_2_/ZnO				98.7		
ZnO				80.7		
SrTiO_3_	NE	10	120	92.1	200	^59^
MnNb_2_O_6_	1/1	3.1985	160	55	90	^45^

a NE: not specified.

The growth of *E*. *faecalis* and *C*. *albicans* was completely inhibited in the presence of NaNbO_3_ at concentrations ≥ 1.25 mg/mL, as evidenced by the absence of CFU compared to the control (Table 2). At the lowest tested concentration (0.625 mg/mL), however, a distinct susceptibility profile was observed. *E*. *faecalis* showed a reduction from 5.81 to 4.20 log CFU/mL, whereas *C*. *albicans* showed only a slight decrease from 3.98 to 3.80 log CFU/mL, suggesting greater susceptibility of *E*. *faecalis* to NaNbO_3_ under this condition. This difference is particularly interesting considering the distinct biological characteristics of these microorganisms. *E*. *faecalis* is a Gram-positive bacterium characterized by a thick peptidoglycan-rich cell wall and considerable ability to adapt to environmental stresses, whereas *C*. *albicans* is a eukaryotic yeast with a more complex cellular organization and a cell wall mainly composed of glucans, chitin, and mannoproteins.^18–20^ Although the antimicrobial mechanism of NaNbO_3_ in the absence of external stimulation has not yet been elucidated, studies involving other materials indicate that microbial inhibition may involve multiple processes, including oxidative stress and disruption of cell-wall or membrane integrity. In *E*. *faecalis*, nanomaterial-mediated antimicrobial activity has been associated with adhesion to the bacterial surface, ROS generation, oxidative stress, and subsequent damage to cellular structures.^17^ Similarly, studies on defect-rich metal oxides have associated oxygen vacancies and redox-active metal centers with ROS-mediated antimicrobial activity. In Gd-doped CeO_2_, for example, the coexistence of Ce^3+^/Ce^4+^ states and increased oxygen-vacancy concentration has been related to enhanced redox activity and ROS generation, with subsequent oxidative stress and membrane disruption.^60^ Oxygen-deficient vanadium oxides exhibited activity against *C*. *albicans*, in which oxygen defects were associated with ROS generation, oxidative stress, and disruption of cell-wall and membrane integrity.^61^ In the synthesized NaNbO_3_, XPS analysis indicated the presence of Nb^5+^/Nb^4+^ species and defect-related oxygen components (Fig. 3), while PL provided complementary evidence for defect-related electronic states (Fig. 4d). Considering the mechanisms reported for other defect-rich metal oxides, these characteristics suggest that surface redox processes may represent a plausible pathway contributing to the observed antimicrobial activity. However, dedicated mechanistic studies are required to elucidate the antimicrobial mechanism of NaNbO_3_.

**Table 2:** Antimicrobial Activity^a^

NaNbO_3_ concentration [mg/mL]	*E*. *faecalis* [Log CFU/mL]	*C*. *albicans* [Log CFU/mL]
2.50	0*	0*
1.25	0*	0*
0.625	4.20	3.80
Positive control	5.81	3.98
Negative control	0	0

a Statistical significance was indicated when *p ≤ 0.05.

Nawaz et al.^62^ reported cytotoxic effects of NaNbO_3_ toward human colorectal carcinoma cells (HCT116), demonstrating effective interactions between the material and biological systems. In addition, pronounced antioxidant activity was observed, indicating that NaNbO_3_ exhibits a chemically active surface capable of participating in redox processes. Evidence of NaNbO_3_ interaction with biological membranes has also been reported by Saranya et al.,^63^ where the material promoted adhesion, proliferation, and migration of MG-63 osteoblast cells, highlighting its surface bioactivity. Although these findings involve mammalian cells and cannot be directly extrapolated to microorganisms, they indicate that NaNbO_3_ can establish interactions with biological environments.

## 4 Conclusions

In conclusion, 1D NaNbO_3_ was successfully prepared by the microwave-assisted hydrothermal method followed by calcination. The material exhibited a polymorphic crystalline structure with rod-like morphology and mesoporous characteristics. XPS analysis indicated the presence of defect-related species, while PL spectroscopy provided complementary evidence for defect-related electronic states. Optical and photoelectrochemical analyses revealed a band gap in the UV region, band-edge positions thermodynamically suitable for the formation of reactive species, and improved interfacial charge-transfer behavior under light irradiation. NaNbO_3_ demonstrated efficient photocatalytic activity, achieving complete MB dye removal, along with good stability over consecutive reuse cycles. Scavenger experiments suggested the involvement of h^+^, ·OH, and ·O_2_^–^ in the photocatalytic process. In addition, the synthesized NaNbO_3_ exhibited effective antimicrobial activity against *E*. *faecalis* and *C*. *albicans*, completely inhibiting microbial growth. To the best of our knowledge, this is the first report describing the activity of NaNbO_3_ against these microorganisms. These findings highlight the multifunctional potential of NaNbO_3_ for environmental remediation and antimicrobial applications.

## Supplementary Material



## Notes

Adsorption experiments and Rietveld refinement (PDF)
