# Supplementary material for: Multifunctional NaNbO3: Photocatalytic Degradation of Methylene Blue and Intrinsic Antimicrobial Activity against Enterococcus faecalis and Candida albicans
Source: ACS Omega. 2026 Sep 16;11(38):57241–9. doi: 10.1021/acsomega.6c04846 (PMC13628632; doi:10.1021/acsomega.6c04846)
Supplement: Supplementary file 1 [file ao6c04846_si_001.pdf]

Multifunctional $NaNbO_3$: Photocatalytic degradation of methylene blue and intrinsic antimicrobial activity against *Enterococcus faecalis* and *Candida albicans*

Daiane Fernandes[a,], Marcelle M. Silveira[b], Thissiana da C. Fernandes[c], Cristiane W. Raubach[a], Janice L. Giongo[b], Rodrigo de A. Vaucher[b], Eduardo C. Moreira[d], Valmor R. Mastelaro[e], Sergio da S. Cava[a,f]*

[a]Post-Graduate Program in Materials Science and Engineering, Federal University of Pelotas, 96010-610, Pelotas, RS, Brazil

[b]Post-Graduate Program in Microbiology and Parasitology, Federal University of Pelotas, 96160-000, Capão do Leão, RS, Brazil

[c]Post-Graduate Program in Engineering and Materials Science, Federal University of São Paulo, 12231-280, São José Dos Campos, SP, Brazil

[d]Spectroscopy Laboratory, Federal University of Pampa, Bagé, 96413-172, RS, Brazil

[e]Physics Institute, São Paulo University, 13560-970 São Carlos, SP, Brazil

[f]Post-Graduate Program in Physics, Federal University of Pelotas, 96160-000, Capão do Leão, RS, Brazil

**Corresponding author: daiane.fg.eng@outlook.com*

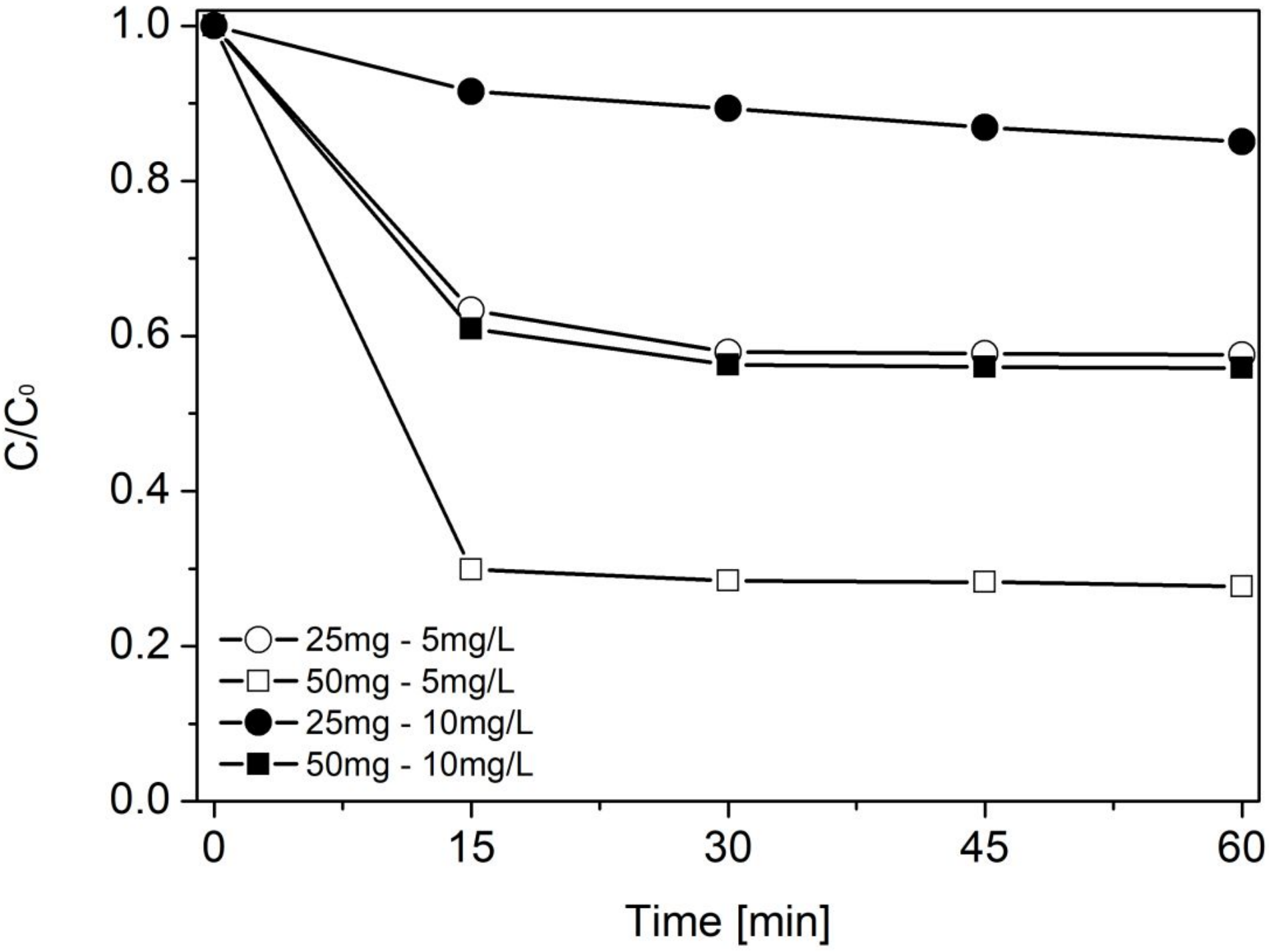

Figure S1: Adsorption experiments.

Rietveld refinement: The Rietveld refinement was performed with the *GSAS-II* software.[1] The peak pattern was refined using a pseudo-Voigt function, while a shifted Chebyshev function was adopted for the background.

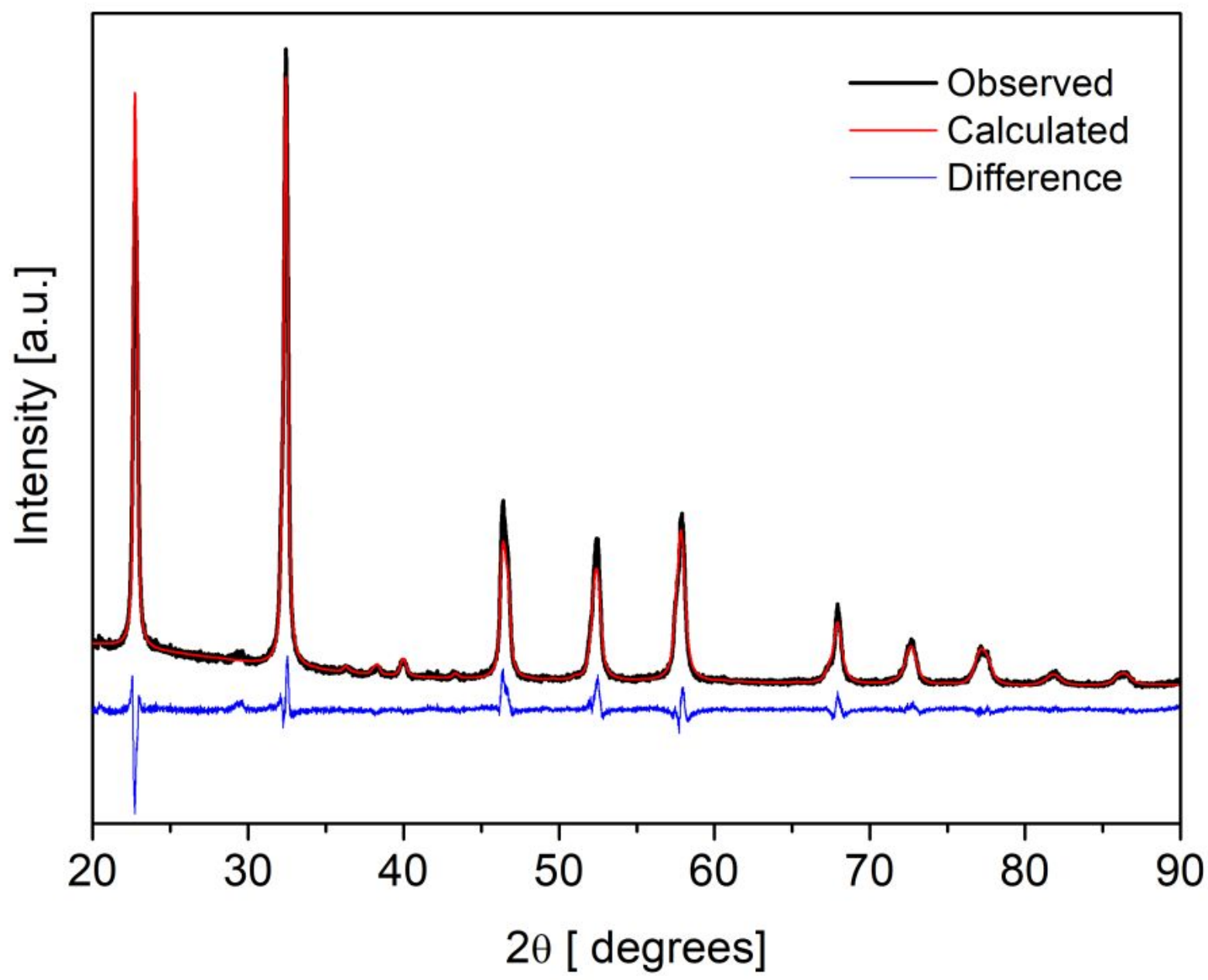

Figure S2: Rietveld refinement.

Table S1: Crystallographic data.

**Parameters**	**Space group P21ma**	**Space group Pbma**
Wt% phase	89.8184	10.1816
a [Å]	5.5447	5.5697
b [Å]	7.7784	15.5240
c [Å]	5.5140	5.5210
Rwp [%]	8.92	
GOF	1.69	
χ^2	2.87	

Reference:

[1] B. H. Toby, R. B. Von Dreele. GSAS-II: the genesis of a modern open-source all-purpose crystallography software package. J. Appl. Cryst. 46 (2013) 544-549. https://doi.org/10.1107/S0021889813003531
